# "The Hospital" Nursing Mirror

**Published:** 1900-05-05

**Authors:** 


"The Hospital" Nursing Mirror.

Being the Nursing Section of "The Hospital."

[Contributions for this Section of "The Hospital" should be addressed to the Editor, The Hospital, 28 & 29, Southampton Street, Strand, London, W.C., and should have the word "Nursing" plainly written in left-hand top corner of the envelope.]

### Notes on News from the Nursing World.

### THE QUEEN'S GIFT TO A DUBLIN HOSPITAL.

The Children's Hospital, Temple Street, Dublin, was specially favoured by Royalty on the day of the Queen's departure. Miss Minnie Cochrane drove up to the hospital in the morning, conveying a gift from the Queen as a memento of the joining of the "Moy-Mell" Children's Guild by the little Battenberg Princess and Princes. The present consisted of a very handsome, large, framed engraving, named "The Queen with her Grandchildren and Favourites at Osborne," and representing the Queen driving in her pony-chair across a grassy lawn, surrounded by the children of Princess Henry of Battenberg. Seven pet dogs also appear in the picture. The Guild, which has been joined by the young Princess and Princes, has for its object the interesting of happy and well-nurtured children in the less fortunate little ones of the poor. The good Sisters of Charity, who form the governing body of the hospital, are often hampered in their work by a lack of clothing for the many ragged children under their care, and are always in want of the many toys without which no children's hospital can be considered complete. The children who become members of the Guild promise, if girls, to contribute two garments every year, made after the hospital patterns; and if boys, to give two toys. Subscriptions are also collected by the various circles of the Guild, under their local secretaries, for the maintenance of four "Moy-Mell Guild" cots. The title of the Guild is taken from the name of a bazaar recently held for the benefit of the hospital. "Moy-Mell" is the Celtic name for the ancient Irish heaven, where all who entered were made young again, and all sickness passed away.

### SIR WILLIAM MacCORMAC ON NURSING AT THE FRONT.

The testimony of Sir William MacCormac as to the value of the work of the genuine nurses in South Africa is emphatic and convincing. He says that it has been "admirable and endless," a meed of praise which really covers all the ground. He continues: "As soon as one batch of serious cases from the front was comfortably settled and a partial rest seemed possible another batch arrived. The untiring energy and patience shown by these ladies under great difficulties and stress have been beyond praise. The sisters of the Army Nursing Reserve, and very many civilian-trained nurses, have, of course, been also employed, and the same remarks, in, perhaps, a somewhat less degree, apply to them also. The members of the Army Nursing Reserve have been trained to a certain extent in army work, but the civilian nurses proper have to be looked after in official matters by the sisters of the Army Nursing Service."

### THE PLAGUE OF WOMEN AT THE FRONT.

Both Sir William MacCormac and Mr. Frederick Treves have condemned the presence of amateur nurses at the front. Sir William says that the work of nursing has "undoubtedly been hampered in some cases by the interference of ladies who know little of hospitals or nurses, and wish, with the best intentions, to help; but their inexperience renders them ineffective for good." This is very mild censure; but Mr. Treves, in his speech at the Reform Club on Saturday evening, was much more outspoken. He said that, so far as the sick are concerned, there are two plagues in South Africa, "the plague of flies, and the plague of women." But while the flies could be got rid of and disappeared at night, the women mentioned in Sir Alfred Milner's proclamation were "an absolute terror." "They come out," proceeded Mr. Treves, "in the guise of amateur nurses, having exhausted every other form of excitement; they take up the time of the officers, and, in fact, have the camp to themselves. Considering the kind of war in which we are engaged, and the number of lives lost, the picture of a number of elaborately-dressed ladies masquerading in summer toilettes and arranging picnics about Cape Town is a blot on the campaign." Although these remarks speak for themselves, and although Mr. Treves definitely mention "amateur" nurses as being those whose doings he was criticising, he has been, strangely enough, supposed to have included "nurses" in his censures, which of course he never did. He has taken the trouble to explain that no such reference was intended, and to emphasise his admiration of "the splendid work done by the nurses." Some further information than has yet been furnished about these amateur nurses seems, however, desirable. In fact, it is only fair to all the ladies concerned that the names of those who constitute the plague should be identified, lest others guiltless of the offence should be tarred with the same brush. A point of even greater importance is, why those ladies whom Sir William MacCormac accuses of hampering the work of nursing by their interference were ever permitted to enter the camp or the hospital. We suspect that if they had not been persons of some social position they would have been politely requested to mind their own business.

### THE WOUNDED IN THE MILITARY HOSPITALS.

Although the War Office authorities have not so far increased the number of fixed meals at the military hospitals, we are glad to learn from the Secretary of State that "unlimited powers" are now assigned to medical officers in hospitals and hospital ships "to add to the fixed diets" whatever extras may be considered necessary in the interests of the sick, and that "it is usual" to give convalescents very considerable additions in the way of milk, fish, porridge, stout, &c., &c. As to the "Princess of Wales" hospital ship, the senior medical officer has "been given full power to make whatever arrangements of meals may be considered most suitable for the patients under treatment on board that vessel." Although the tea hour varies, the officers in charge of military hospitals have imposed upon them the obligation of seeing that "patients are not left without nourishment between the tea hour and the breakfast hour."

### THE NURSES ON THE "MAINE."

THE "Maine," which has started on her return journey to the Cape, has had no female nurses on board. Speculations have been freely indulged in as to the reasons why the services of Miss Hibbard and the other lady nurses have been dispensed with. We need scarcely say that the question of comparative competence is not involved. Miss Hibbard and the four sisters who were her colleagues are among the most accomplished of American nurses. The fact of the matter is, we understand, that, it being found that the nursing staff was in excess of the requirements, it was considered advisable, in view of the importance of economising space in the vessel, to entrust the whole of the duties to males. Of course, the training of the latter is indisputable, and they are quite qualified for the work which they have undertaken.

### THE "CORPS OF LADY HELPS."

A FORTNIGHT ago we warned our readers that the corps of lady helps which the Duchesse di Villanda aspired to organise to aid the wounded at the front would not, in any case, be allowed to proceed there. It now appears extremely unlikely that the corps will be formed. The lady who originated it claims that her title is a Spanish one, but the Spanish Ambassador in London is not aware of its existence, and until the matter of personal identity and antecedents, with some other points of interest, have been settled, it is scarcely likely that the St. Patrick Sisters will proceed on their expedition. Even if an English duchess, known and respected by all classes, proposed to take out a band of women without official authority, the movement would merit reprobation. There are already ladies of title enough and to spare at the front.

### PRINCESS CHRISTIAN'S TRAINED NURSES.

THE thirteenth annual report of Princess Christian's Nurses, for 1899, shows that during the year thirteen past and present nurses belonging to the Home at Windsor and on the Army Nursing Service Reserve have been called up for duty—nine for hospital work in South Africa, two on the "Princess of Wales" hospital ship, and two for home military hospitals. Two district probationers have received training in district nursing, and one was enrolled as Queen's nurse. The district nurses nursed 442 cases in Windsor, 505 in Eton, 101 in Egham, 157 in Addlestone, and 90 in Chertsey. Miss Edden, who became assistant superintendent in March, has been appointed Lady Superintendent of the Nottingham and Notts Private Nursing Institution.

### NURSING IN HOSPITAL WITHOUT REGULATIONS.

FROM an interview, which appears in another column, it will be observed that at the German Hospital, Dalston, the nursing is carried on without printed regulations. Of course, in many respects the system of the religious orders, whether Lutheran or Romanist, differs from the system in this country, but it is curious that regulations should be entirely dispensed with, even in an institution with a small nursing staff. Apparently, in this case the absence of them does not interfere with the preservation of discipline. There are, at any rate, fixed hours for meals, which are served five times during the day; and though off-duty can only be secured when there is no pressure of work, regular holidays, varying in length, are given. On the whole, the nurses at the German Hospital do not fare badly.

### ST. GEORGE'S UNION INFIRMARY.—A WITHDRAWAL.

THE attempt of the Guardians of St. George's, Hanover Square, to compel the members of the staff to sign a form of consent to a medical examination, whose provisions we set forth in our issue of March 10th, has, we learn, proved a failure, and is to be dropped. We congratulate the staff upon the result of their hostility to an innovation that ought never to have been entertained, which might not have been so satisfactory if publicity had not been given to the matter.

### WORKHOUSE INFIRMARY NURSING.

IN the twentieth annual report of the Workhouse Infirmary Nursing Association there occurs the following passage: "It is at last admitted without question that trained nurses are a necessity in workhouse infirmaries, and the point to be considered is how best to meet the demand. We can only reiterate what we have said from the commencement of our work, nearly 20 years ago, that so long as there is no fixed policy, no recognised standard for one and all infirmaries alike, and no secured position for nurses, we shall still hear of unsatisfactory conditions in our infirmaries, both large and small. The committee would be glad to see a strong committee of guardians formed who would steadfastly determine to take up a matter that would affect their administration so closely, and which clearly demands unity of action amongst those whom the question most concerns." The suggestion is an admirable one. The association has been able, as the result of circulating a number of questions among the boards of guardians, to ascertain that while on the whole there is a lack of unanimity amongst them, and a failure in most cases to grasp the paramount difficulties, there are many guardians who are anxious to maintain a high standard of efficiency in their infirmaries. From among the latter it should be practicable to constitute the kind of Committee proposed. The need of unity of action is constantly being manifested, and the association report that evidence is "daily" brought to them of the increasing difficulty boards of guardians are having in securing the services of trained nurses. The association have for many years supplied nurses to the Eastry, Hastings, Newton Abbot, Plymouth, and Wilton boards, among others, but this year they have been unable to do so, as by degrees their staff of nurses is becoming reduced. This state of things will continue until the conditions of employment are largely altered, and until a general regulation for all unions, either making workhouse nurses responsible to the medical officer and not to the master or matron of the workhouse, or providing for the appointment of a trained matron, is enforced.

### THE MARCHIONESS OF LONDONDERRY ON DISTRICT NURSING.

THE wife of the new Postmaster-General takes a practical interest in district nursing. Presiding at the seventh annual meeting of the Comber District Nursing Society, Lady Londonderry said she was greatly in favour of trained nurses, and that her experience during the past year had taught her the need of them. She has always been, however, the society's greatest help and support. It was by her encouragement that it was formed. Last year Nurse Fitzsimons made upwards of 2,000 visits and attended 130 cases. The satisfactory financial condition of the society is attested by the fact that the treasurer's report showed a balance in hand of £52.

### NURSING IN JAPAN.

Two nurses were lately sent out to Japan by the Colonial Nursing Association. One of them, writing to Mrs. Francis Piggott from Tokyo under date of February 11th, gives some interesting details of their reception: "I am sure you will be glad to hear that we are very comfortably settled in our new work and home. We had a delightful voyage out on board the 'Sachsen,' and we were very well looked after. The first part of the trip was not quite as nice as the last, for we were very crowded in our cabin. There were four of us, and you know what that means when two out of the four are very sick; and it was not pleasant in the Red Sea! However, after Colombo we were alone, and then we soon forgot the discomforts we had gone through. We were met at Yokohama, and 'A Warm Welcome to Japan' came by the N.D.L. agent before our ship got in port. We are living in a charming little Japanese house all by ourselves; we have a Japanese woman to look after us, and one of the British Legation gardeners comes to sleep in the house, so that we have a man on the premises. Our arrival had been most anxiously looked for. An American lady was expecting her first baby, and she was terribly afraid we should not arrive in time. My colleague packed up the same evening we arrived in Tokyo, and after all the baby did not come for more than a week. I have been to two medical men in Yokohama; was telegraphed for the second case before I was free from the first. The doctors have all been most kind to us, and they say we shall have plenty of work. Very nice arrangements have been made for our board when we are at home. The Japanese women just buy everything we want, and the bills go to the Legation. Our washing, too, is all paid for, and inquiries are being made with the view of securing the insurance of our belongings, which is necessary, because there are so many fires here. The people are wonderfully kind."

### THE LEICESTER TRAINED NURSES' INSTITUTION

It appears that some remarks headed, "Pay up, Leicester," made by us on April 14th, in reference to the Leicester Institution for Trained Nurses, have been curiously misunderstood. The Lady Superintendent of the private nursing branch of the institution has received letters of sympathy in respect to a position which does not exist. Our allusion was only to the district branch, and, as the chairman of the institution informs us, as thus applied it is entirely correct. The finances of the private nursing branch, on the other hand, are perfectly satisfactory; so much so, that the committee have been able this year to present their nurses with a substantial bonus for the third year in succession. We hope that before another report is issued the district nursing branch will also be placed on a satisfactory financial basis.

### THE MARY WARDELL CONVALESCENT HOME FOR SCARLET FEVER.

In the annual report of the Convalescent Home for Scarlet Fever, the committee draw the attention of the subscribers to the great need of wider general support of this excellent institution. As a means of providing urgently-required additional funds to pay for essential expenditure in repairs and a new system of drainage, a concert will be held next Tuesday afternoon, by the kindness of the Duchess of Sutherland, at Stafford House. The income of the home barely suffices for the ordinary current expense, and it has, therefore, been impossible to accumulate any reserve. Those who buy tickets for the concert will be helping a good cause, as well as affording themselves the pleasure of listening to the clever artists who have given their services. The work of the home was last year described in detail in these pages, and its usefulness is demonstrated by the single fact that since it was opened in 1884 no less than 3,274 patients have been admitted.

### NEWCASTLE NURSES' HOME AND TRAINING SCHOOL.

The twenty-eighth annual report of the Newcastle-upon-Tyne Nurses' Home and Training School is in all respects but one satisfactory. There was, unfortunately, more sickness among the nurses last year than during the previous ones, but notwithstanding this, the committee have been able to give the nurses bonuses amounting to £361, in sums varying from £2 to £10, according to the length of service. The staff now numbers 80 trained nurses and seven probationers, and the cases nursed during the year are divided as follows: Medical, 385; surgical, 73; maternity, 66; infectious, 92; and mental, 26. Twenty-six nurses were employed in various hospitals and infirmaries, and two have left the home in order to nurse in South Africa. The home is now entirely self-supporting, any donations sent being reserved for the nurses' sick fund. Last year the nurses' earnings reached the handsome amount of £3,984, and the balance in hand was £219.

### SHORT ITEMS.

In the absence of the Countess of Derby, the foundation-stone of the new Nurses' Home at Blackburn was laid on Thursday by Mrs. Baynes, the wife of the High Sheriff of Lancashire, one of the founders of the Nursing Association. The home is to provide accommodation for 12 nurses and a superintendent, and will cost, with the site, from £5,000 to £6,000. — The annual meeting of the Association of Asylum Workers will be held at the Medical Society House, 11, Chandos Street, Cavendish Square, on Monday, the 14th, at four p.m., Sir James Crichton-Browne, the president of the association, in the chair.—As a mark of deep sympathy with England in the present period of anxiety owing to the war, the committee of the Italian Hospital have resolved to abandon the usual festival ball this year, but they trust that friends and supporters will not allow the charity to suffer on this account.—Mary Beatrice Manning met with a richly-merited fate at the Old Bailey on Monday. Her frauds on nurses, which have been described in these columns, were only incidents in a career of crime extending over thirteen years, and she has been sent to penal servitude for five years.—At the Oxford City Quarter Sessions last week, Marie Thompson was sentenced to six months' hard labour for obtaining money under false pretences. This is the individual who was known for a time at Osney as "dear nurse," and whose freaks of imagination were mentioned in our issue of April 21st.

### Lectures on Medicine to Nurses.

By H. A. Latimer, M.D. (Dunelm), M.R.C.S. Eng., L.S.A.Lond.; Consulting Surgeon, Swansea Hospital; Past President of the South Wales and Monmouthshire Branch of Brit. Med. Assoc.; Past President of the Swansea Medical Society; Hon. Life Member of the St. John Ambulance Association, &c.

### VII.—THE ANTIPHLOGISTIC TREATMENT—HUMORAL PATHOLOGY.

As a result of theorising, unfettered by true knowledge, a work written on the subject of the practice of medicine would be found to be littered with now discarded opinions as to the causes of disease and the modes of treatment to be adopted for curing the sick. Some of these treatments were more harmful to the invalids on whom they were practised than were the maladies they were instituted to combat. Thus, for instance, under erroneous conceptions of the cause and nature of illnesses accompanied by fever, a treatment known as the Antiphlogistic system was practised with, I doubt not, much injury to those subjected to it. The word Antiphlogistic is derived from the Greek "anti," opposed to, and "phlogistos," burnt. In one sense any treatment which has as its object the reduction of fever heat and excitement during inflammatory attacks is an Antiphlogistic one, but the system I am discussing had its especial methods, which were absolutely opposed in character and theory to those which obtain in these days of science. Regarded from the modern point of view, it was faulty in conception, and was most inimical to the patient's welfare. I grant that it was the best that could be practised at a time when little was known of the true nature of disease, but as it was based upon the treatment of symptoms, and not upon their causes, it was very mischievous. It aimed at reducing body heat, excited action of the heart, mental excitement, and all the other phenomena of the fever process by blood-letting, a lowering diet, and by the use of medicines which had strength-reducing properties. If the illness which the physician was called in to combat was of a quickly-terminable nature—such, for instance, as an inflammatory cold—these remedies undoubtedly did good; but if it happened to be of a character which we now know had to run a definite and prolonged course, the patient's chance of recovery was seriously lessened from the start, for he was then compelled to face a lengthened period of disease which, under any circumstance, was sure to be of an exhausting character, with his strength sapped by a strength-reducing treatment at the very start of his illness. You would stand aghast in your present state of enlightenment were you to read works of men who practised during these days, and would wonder how sick people ever recovered at all from grave illnesses. I will quote the observations on treatment by one of the greatest London physicians, written about 1845. The writer, Dr. Latham, is one whose works will justly stand as memorials of classic writing. They are most interesting reading, both from the beauty of their style and from the evidence they bear of thought and experience on the subjects with which they deal. They are also very philosophical in their reflections upon the different measures to be adopted for curing disease. I will quote, for instance, three paragraphs of his when he is discussing the treatment of acute rheumatism. He says: "I have seen people enormously bled in acute rheumatism, and their entire disease swept away at once, and health restored rapidly. And the practice which will do this, is it not a splendid and a tempting practice? Again, I have seen people enormously bled in acute rheumatism, and their disease swept away at once; but they have forthwith gone raving mad. And a practice which will do this, is it not a hazardous practice? And, again, I have seen people enormously bled in acute rheumatism, and no single pain has been mitigated; but the disease has continued for an unusually long time in its acute, and then has degenerated into its chronic form. And a practice that has this issue, is it not a doubtful practice?"

In another part of his work he gives the case of a man who was under treatment for acute rheumatism, and who underwent the *régime* of the day at his hands. As inflammation of the lining membrane of the heart (the pericardium) set in, he was bled and salivated. I give his own words: "The patient being a strong man, and his pulse being now more full and hard, and his fever more fully developed, and a vital organ inflamed, was bled by venesection to twenty ounces, and ordered three grains of calomel, and a quarter of a grain of opium every three hours. The next day the general symptoms being the same, and the exocardial murmur unaltered, he was bled again by venesection to eight ounces, and four ounces more were taken by cupping from the præcordial region, and the calomel and opium were still continued." The next day he seemed a little better, so nothing was done. "The next day the fever continuing fully developed and the pulse full and hard . . . ten ounces more of blood were taken from the arm." Four days later on eight leeches were applied over the region of the heart, and the calomel and opium were given again. The case terminated fatally in the end.

Now this case is a good example of the many which were treated on similar lines almost to within my memory. Nothing was really known as to the life-history of disease by the profession of medicine at large until men took to placing the sick under the most careful observation, and with every advantage as far as diet and nursing was concerned, but with their progress, when doing well, unaffected by the administration of drugs. This treatment, by which so much true knowledge of the progress and natural terminations of disease was obtained, is called the "expectant treatment," and is not by any means of quite modern introduction. It was practised by Dr. Sydenham, "the Father of English Medicine," as early as 1666, but evidently obtained no hold upon men's minds as a definite procedure at that time, if we may judge by the severe regimens of diet and medicines to which invalids were subjected until quite recently. Even now there is a tendency to too much drugging. The public insists on being treated with medicines during sickness, and finds it difficult to believe that cures can result by means of regulated diet, suitable hours of work, and the breathing of pure air of a suitable character for the case under review.

Passing from this subject of the antiphlogistic treatment, I will speak shortly upon two other theories of the treatment of disease which for a time dominated men. The treatment in vogue at any period is necessarily the result of the view held at that time of the cause and nature of disease. Hence it arose that when men believed that disease was due to morbid states of the fluids in the body—an opinion which prevailed when the so called "Humoral Pathology" held sway in men's minds—all efforts were made to deal with these humors themselves, the solid organs which produced them being ignored and left alone. Then issues and setons were in vogue, and people kept running sores on their limbs as safety valves for the escape of fluids which it was thought would injure them if retained in the body. Just think for a minute of such beliefs in our present days of enlightenment! Imagine anyone submitting to having an artificial wound made and kept open on the neck, arm, or leg, for the purpose of warding off some impending illness! You will find a relic of this belief in the minds of ignorant people when you are told, as is often the case with such folk, that they do not like you to heal an ulcer on the leg, as they fear, by your doing so, that their health will suffer. This is an undefined survival of the humoral pathology of the past.

### Nursing at the German Hospital.

### A CHAT WITH THE MATRON.

By Our Commissioner.

Following the celebration of the fifty-fifth anniversary dinner of the German Hospital on Friday I visited that institution on Saturday for the purpose of having a talk with the matron about the nursing system. The hospital, which stands well back from the main road in Dalston Lane, and has a Lutheran church for a very near neighbour, is a building of considerable dimensions, with pleasant grounds for the use of the patients who are able to get out. Accompanied by one of the sisters, I made an inspection of several of the wards, which are exceedingly light, airy, and well arranged. Bath-rooms are in convenient proximity, and the operating-rooms are quite up to date. It was pleasing to notice how the worn faces of some of the sufferers brightened at the entrance of the sister, while in the children's ward she received a more demonstrative welcome. The matron has only been in her present position since last October, but she had previously been a sister at the hospital for nearly six years.

### The Nursing Staff.

"And how many sisters are there?" I asked.

"Fifteen, and five male attendants for the men."

"Then all the nurses are called sisters?"

"Yes; we have none of the distinctions that are observed in English hospitals, such as probationers, staff nurses, and night superintendents."

"But the sisters are trained?"

"Oh, yes, all of them in Germany, at the Deaconesses' House, Bielefeld, where there are generally upwards of eight hundred nurses."

"What is the age of admission to Bielefeld?"

"No one can be admitted who is under eighteen, but there is no other condition."

### The Training and the Vow.

"How long is the period of training supposed to last?"

"There is no specific time. Some are in training for four or five years. They all have to learn everything. One great feature at Bielefeld is the number of epileptic patients. There are also usually nearly a hundred children, so that the sisters get to know how to nurse children as well as men and women. The vow is not taken for five years until the sisters have had an opportunity of seeing whether they really like the work."

"Then yours is a religious order?"

"Distinctly, and a clergyman is at the head of it, but of course it differs entirely from the Roman Catholic orders. We can leave if we like and we feel that we cannot with advantage go on with the work."

"Then what is the precise nature of the vow?"

"We promise to stop as long as the Lord thinks it good for us to remain. When we take this vow we are supposed to have resolved to give up our whole lives to nursing the sick, and as a rule the resolution is faithfully adhered to. Very few sisters leave the order. The brothers take a similar vow."

"You mean the male nurses?"

"Yes; they are as well qualified to nurse as the sisters, though they only work in the male wards. There are no brothers in this hospital."

"What is the lady head at Bielefeld called?"

"The Mother. She sends the sisters just where she pleases, chiefly to various hospitals in Westphalia, though some go to Paris, Brussels, and Antwerp."

### The Sisters' Duties.

"How would you describe the duties of the sisters here?"

"They give medicines, take temperatures, change the bandages, make the beds, and do a little dusting."

"But there are ward-maids?"

"Only in the women's and children's wards."

"What are the hours on duty?"

"The day sisters start at half-past six in the morning, and generally finish at eight in the evening; but if necessary they remain until ten. There is no stated time for off-duty. A sister goes off when there is nothing special to be done, and if many patients are very ill she does not go off at all."

"And the night sisters?"

"Lately two sisters have been on duty at night. They begin at ten p.m., and stay until six in the morning. There is a male attendant on duty in the male wards during the nights. Accident cases often come in during the night, and they are attended to by one of the doctors and the sister. We have three house doctors. Accidents are a great feature at this hospital."

### No Regulations.

"What are the rules about holidays?"

"The sisters have a fortnight's holiday one year, and a month's the next. When they have a month they usually go to Germany, and when they have a fortnight to the seaside in England. We have no printed regulations about anything. Everybody knows the hours for meals. We begin the day with prayers at seven, and end it with prayers at nine. They are always said in the dining-room. The servants attend as well as the nursing staff. As to the hours for meals, breakfast is at seven, lunch at nine, dinner at half-past twelve, coffee at three, and supper at half-past seven."

### Sisters on an Equality.

"Are the sisters of different grades?"

"No, all are on the same footing. If one leaves the matron arranges as she likes. Sisters are liable to be called away to Germany. They are not obliged to stay in London for any particular period. If they want to return to Germany they tell me, and I arrange accordingly. If a sister is not able to discharge her duties here, from any cause whatever, she is sent back to her native land."

"Is there any difficulty about language?"

"We have a good many accident patients in who are English, but several of the sisters speak English. Though the hospital is primarily intended for Germans and persons who speak German, other patients are not refused if we have room. A great many of our numerous out-patients are English."

### Pensions not Needed.

"Is there any age limit in operation?"

"The sisters go on working as long as they are able. When they are past work they go back to Bielefeld, and are maintained there. After they have taken the vow, they are kept in the houses till they die."

"Others than Bielefeld?"

"Yes, there are about fifty Deaconesses' Houses. The first matron here, Miss Christine Burger, and the three or four sisters who accompanied her, came from Kaiserswerth. She was matron for upwards of fifty years."

"You have a convalescent home and a sanatorium? How about the sisters there?"

"There are two additional sisters in the convalescent home. One of them has been here more than half a century. Most of the sisters came six years ago."

"Your system is very different from the English system. Have you any general remarks to make about it?"

"I know that there are many merits in the English system, but I like ours better. Certainly we attach much importance to training, and hold as strongly as you do that only persons who have been taught to nurse properly should attempt to undertake the work."

### Nursing the Wounded on the "Austral."

### A TALK WITH COLONIAL NURSES IN LONDON.

By a Special Correspondent.

"It's by the merest chance," said Nurse Elizabeth," that I have Nurse Maud with me in these lodgings. Without her I should be terribly lonely."

The two, Nurse Elizabeth and Nurse Maud, had just arrived in London in part charge of about 200 sick and wounded soldiers from the front. It seems a pity that the nurses, after landing their patients safely on shore, should be as it were turned loose themselves, unless, as is the case with some, they have friends to go to. Nurse Elizabeth and Nurse Maud were, I thought, feeling just a little lonely, and a trifle inclined to take the next boat back to the Cape. So the first thing we did, when they had told me how Nurse Maud, not finding her sister as she expected, had arrived at the lodging late on Wednesday night, was to get out a map of London. On this we traced the way to several places they were anxious to visit while they are in town, one of which was the Herbert Hospital at Woolwich, where they wanted to see the men they had nursed on the voyage. One can hardly realise the difficulty the mere getting about is to those who have never been in England before, and who suddenly find themselves set down in the largest capital in the world without a guide.

"You are very much behind us in some ways," they said. "You have no electric light, and they tell us you have not yet begun to use electric trams; we have both at the Cape."

I hastened to put them right on these points, and in return asked for enlightenment as to some of the reports that have been going about in the papers as to the state of civilisation in South Africa. "For instance," I asked, "is President Kruger such a rough and uncultivated person as he is represented?"

"Not at all," Nurse Elizabeth answered. "I know him quite well; he is extremely particular, and always goes to the Government House in great style. The newspaper accounts are very far-fetched."

"Is it true," I inquired, "that one of the nurses with you on the 'Austral' was a cousin of his?"

This highly amused them both, for it was the first they had heard of it. "I think it must be myself," laughed Nurse Elizabeth; "I was somehow taken for a relation of his, but it is perfectly untrue."

### A Beautiful Voyage.

I had been rather puzzled by being told that all the nurses on board the "Austral" were South Africans; and the nurses explained that the correct way to describe themselves is as British Colonials. Both are of English or Scotch parents, but they are colonial born and bred. Nurse Elizabeth was trained at Wynberg Hospital, eight miles from Cape Town, and Nurse Maud at Kimberley.

"Then are you army nurses?" I asked.

"No; we were both engaged in civil hospitals, but every available place now is crowded with soldiers, so we have had a good deal of experience in army work."

"And you had a pleasant voyage home, I hope?"

"Very pleasant, indeed. Have you seen the 'Austral?' She is a magnificent ship, splendidly fitted up, and the accommodation was excellent. Should you like to hear how we arranged the work on board? You know there were four of us, and Sister Ethel was in charge, so she did the rounds with the doctor, a very clever man, who has just gone to the Herbert Hospital. Two of us were on day duty with Sister Ethel, and one at night. The large saloon was converted into a temporary ward, and here the men were placed, of course in swinging cots. The orderlies had charge of the mess, but of course we attended to the low diet ourselves. The food was first-rate; we had only to ask for what we wanted, and it was supplied at once. The men were all so nice and grateful, too; and so cheerful, it was quite a pleasure to nurse them. In fact, we had a delightful time altogether; the voyage was a real pleasure trip."

"Was it very hard work?" I asked. "At any rate, you were better provided as to staff than the 'Winifredian,' for instance, which had only two ladies in charge, one of whom was taken ill herself on the voyage."

"It was hard, because there was so much running backwards and forwards. We were all the time fomenting! I never saw so much ear-ache and eye-ache as there was; I think it must have been from sleeping in draughty tents. Our cases were mostly medical ones—fever, dysentery, and one typhoid. He died, poor fellow, though we and the doctor did our very best for him."

### A Funeral at Sea.

"Did he die before you reached land?" I asked.

"Yes. We arrived on Wednesday, and he died on the Sunday before. I shall never forget what a touching sight it was. We did everything just as we should for a funeral on shore, and covered him with the Union Jack. The Captain read the Church Service; and the only difference was in the words, 'We commit his body to the deep,' when the body was slid off the side of the ship. The engines were stopped, and we came slowly past, leaving him there."

### About the Cape Hospitals.

"There was only one case that I thought ought not to have been moved so soon," Nurse Elizabeth went on. "It was that of a man suffering from enteric fever, and he was very ill indeed. But we got him safely to Woolwich. The papers are wrong in saying that only one man was unable to walk; we had six cot cases."

"You had some very serious surgical cases, too, had you not?" I asked.

"Yes. One was shot through the mouth. The bullet came out through his neck. But he is getting on quite well now. Now do ask me some more questions; I don't think I've told you anything yet. What would your readers like to hear about?"

"I think," I said, "that you have told me a good deal. But as our readers are mostly nurses themselves, perhaps they would like to know what the Cape hospitals are like; whether, for instance, the training lasts three years, as it usually does in England?"

"Yes, just the same. At the end of the time we either stay on in the wards or else do private nursing."

Then I inquired whether they had many coloured people to nurse.

"Quite as many coloured people as white ones—Zulus, Basutos, and Kaffirs—there are such numbers of them in Cape Town, you know. Perhaps you don't know, though, how strict the laws about the coloured population are. For example, they may not walk on the pavement, but only in the road; and in the Transvaal no coloured man may be about in the towns after nine o'clock, and if he is found without his passport in his pocket he is taken off to prison."

"Has he," I gently asked, "a pocket?"

"Oh dear, yes. They are very fine gentlemen. Personally, I think these laws quite necessary, for they are extremely insolent in their behaviour. But the Zulus speak a beautiful language—it is so musical."

Nurse Elizabeth proceeded to tell me something of the terrible divisions in families that have been brought about by the war; her own sister, having married a colonial with burgher rights, is, as far as she knows at present, nursing Boers in the field, the husband having been commandeered.

A Baby Shot in the Field.

"There are lots of Boer women and children in the field," Nurse Elizabeth went on, "and the other day a Boer baby was brought into hospital with a gunshot wound. They all learn to shoot, boys and girls alike, as soon as they can handle a gun."

These nurses have six months' leave before going back to the Cape; and I expressed the hope that they would have a really good time while they are in England, and see as much as they would like of the old country.

### The Nurses of the Imperial Yeomanry Hospital.

Writing from Deelfontein, March 31st, a correspondent says: It seems scarcely possible to believe that we have already been settled here for a fortnight, and so much has happened in this short time that I feel it rather a difficult matter to know where to begin relating to you a few of our experiences—pleasant and otherwise—since leaving England, just six weeks ago. Our voyage out was quite delightful and enjoyable, that is to say after we got out of the Bay. Our experiences and appearances in that proverbially stormy little quarter of the briny ocean I much prefer not to describe. Suffice it to say that even our good captain of the "Guelph" was, I am quite sure, very glad to be the other side, for the poor man had been enjoying some very solitary meals in the saloon those few days when we were tossing about in the Bay. You have heard already how we managed to kill time on board ship, and how we made the most of the few hours we had on shore at Teneriffe and St. Helena.

Results of the Storm.

When we arrived at Deelfontein, after about thirty-six hours' travelling, the heat was intense; but there was no sign then of the storm which we experienced a few hours later. Certainly those who had preceded us a week or ten days had not been letting the grass grow under their feet, for we found huts and tents enough to accommodate 200 patients already erected, besides our own sleeping accommodation, which consisted of a large marquee to hold 20 beds and two smaller huts, each holding 10 beds. These latter will eventually be used as wards. Since the collapse of our marquee, which has been described in a previous number, we have all been moved to a large hospital hut pending the building of our own small houses. On the same day, and almost at the same time, our mess tent also was blown down, and two or three times since, generally managing to collapse when the sisters are taking their meals. The only thing to do then (to ensure finishing your repast) is to pick up your plate of food and run outside on the veldt to eat it. Anything left on the table is of course very soon swept into the dust. These slight inconveniences do not happen very frequently, however, otherwise the patience of the cooks and those who cater for us would be tried to the utmost limit.

Our Surroundings.

And now a word about our surroundings. This camp is situated under a range of kopjes facing the railway line, which is a stone's throw in front of us. To the right is the open veldt, or, I believe, properly speaking, the "karoo." To our left is a Kaffir kraal, and beyond that again the station, and a camp of some 50 odd men of the Duke of Edinburgh's Own Rifles, who are placed here to guard the line of communication and our camp. The hospital block is in the centre of the camp, with the doctors' tents on the left and the sisters' encampment on the right. The dressers and orderlies are accommodated in tents on the opposite side of the line. These huts are built of corrugated iron lined with wood, with a door at each end, and the windows are made like ventilators with slanting shutters outside, and there are five down each side, so that plenty of light and air is received.

Provision for Convalescents.

Besides the huts, which number about ten at present, tents are erected, tortoise and bell shaped, fitted up for the use of the more convalescent patients, mild cases of disease, and small surgical cases. They vary in size, the larger ones containing eleven beds each. Building is still progressing fast, for we are full up, and we want to be ready to take another hundred patients in by the end of the week. It is difficult to realise sometimes that we are not working in smart London hospital wards, for every little detail connected with the comfort and well-being of Tommy, and calculated to ensure him a speedy recovery under the most favourable conditions, has been thought out and arranged for. Each hut is provided with iron bedsteads, spring mattresses, and beautiful sheets, blankets, and feather pillows, and a bedside table and chair are assigned to each patient. Outside the wards are comfortable lounge chairs with cushions for the use of those men who are able to be moved out. We have a great number of typhoid patients, some very severe cases; but I am glad to say that so far we have not lost any.

The Question of Food.

The obtaining of suitable food for so many mouths causes no small amount of trouble to those in charge of the commissariat. Meat has to be got from Cape Town; milk from the neighbouring farms is difficult to obtain, and costs 3s. a quart; eggs are almost unknown, and fruit is a great luxury. Of the latter we have only seen a few grapes and some small green figs.

"Windsor Castle" and "Curzon Home."

The convalescent soldiers are vieing with each other in making smart gardens outside their tents. The sand is carefully banked up, white stones used for a border, and plants from the surrounding veldt and kopjes, principally the amaryllis lily and a prickly sort of heath, are stuck in, and the name of the cottage marked out in little white stones. Already we have "Windsor Castle," "Paradise," "Devonshire House," "Tommy's Rest," "Cottage of Content," "Villa de Fripp," and "Curzon Home," and some original men have on one side of the door "God save our Queen" and on the other "We are happy," while the Prince of Wales's feathers flourish all over the camp. Next week I hope to tell you something of the patients personally, and a few of their thrilling experiences since they were ordered south.

### Death in Our Ranks.

We regret to record the death of Miss Dorothy von der Goltz, which occurred on Wednesday, April 25th, from pneumonia and purulent pericarditis. She was trained at Chichester Hospital, and had held several positions as district nurse and matron, notably that of matron of the Cancer and Skin Hospital, Liverpool. Lately she has been working at the Nursing Institute at Cricklewood. Any communication should be addressed to the Matron of the Nursing Institute and Medical Home, Claremont Road, Cricklewood, N.W.

### Royal British Nurses' Association.

### MEETING OF THE COUNCIL.

A meeting of the General Council of the Royal British Nurses' Association was held at 11, Chandos Street, Cavendish Square, on Friday afternoon. Dr. Godson took the chair, and among those present were: Mr. Langdon, Mr. Fardon, Mrs. Dacre, Mrs. Coster, Miss Thorold, Miss Wedgewood, Miss De Pledge, Dr. Bezly Thorne, Mr. Gant, and Miss Hutchinson.

After the minutes of the previous meeting had been read and confirmed, the hon. treasurer presented the report for the year ending March 31st, 1900. It shows that there has been an increase under all but two headings, thanks to the sound financial basis upon which the society has been established by the Café Chantant last year. The deficits are on the conversazione and the journal accounts. The former is explained by the printing of tickets, &c., and the latter by the increase in the size of the journal, which augments the cost of production by £4 per issue. They will be covered, it is anticipated, by advertisements which have recently been secured. The "Helena" Benevolent Fund has proved very useful. Grants to the extent of £63 have been made, and there is a balance of £29 in hand. This fund has been loyally supported by the members of the Association, but the treasurer recommended it to the generosity of the public, as the earnings of nurses were too slender and precarious to cope with all the cases needing help. The expenditure this year was £955 19s. 8d.; the receipts, including the proceeds of the Café Chantant, £2,170; leaving a credit balance of £1,214.

Miss De Pledge seconded the adoption of the balance sheet, which was agreed to.

Mr. Fardon read and commented on the report of the executive committee. During the last three months 31 nurses have been registered, 39 new members have been elected, five members have withdrawn, and four have died. The association has lost two distinguished vice-presidents—Sir Thomas Grainger Stewart and Sir Douglas Maclagen. Sir William Priestley's death has also deprived it of a valued member and friend. A communication from Dr. Peel Ritchie states that the members of the Scottish executive committee do not see their way to appointing a successor to Dr. Calder Leith, late hon. secretary of that branch. There are several difficulties in connection with the Scottish branch of the association which make it necessary to consider its reorganisation. The committee, considering the admission of mental nurses to the Association, have deferred their report pending the perusal of further inquiries. References to the recent sessional lectures, the issue of the roll of members for 1900, the reprint of the charter (to be obtained by members at a cheap rate), the new cards of membership, completed the report.

Dr. Bezly Thorne seconded the adoption of the report, which was then put to the meeting and carried.

Mr. Fardon moved, and Dr. Bezly Thorne seconded, that the regulations for Colonial branches passed at the meeting of January 26th, 1900, be confirmed.

Dr. Bezly Thorne proposed that a vote of thanks from the members be conveyed to Mr. Sidney Spokes, Mrs. Scharleib, M.D., Miss Mabel Anderson, and Mr. Richard Greene for their lectures, which have been highly appreciated and well attended.

Miss Hutchinson proposed that a settlement providing free quarters for aged nurses be established, and Miss Wedgewood having seconded, it received the cordial support of the meeting.

A vote of condolence to Mr. Gant upon the death of his wife was also agreed to.

### LECTURE BY MR. RICHARD GREENE.

The last meeting of the session of the Royal British Nurses' Association took place on Thursday evening, when Mr. Richard Greene, F.R.C.P., late superintendent of Berrywood Asylum, delivered a striking lecture on "The Insane, the Asylum, and the Nurse." Sir Henry Burdett, K.C.B., occupied the chair, and in introducing the speaker said that Dr. Richard Greene was the oldest superintendent of lunatic asylums in the whole country, and that he was the first to recognise the necessity of trained nurses in those institutions. At Berrywood a probationer can be thoroughly instructed as a nurse and prepared for her duties in every way.

Dr. Greene's lecture commenced with an exhaustive history of mental disease and its treatment since its earliest records. Before the Christain era lunacy, said the lecturer, was receiving scientific study and treatment, but with the dark ages, when its cause was attributed to possession by an evil spirit, all remedies were retrograde. Pity, goodness, and justice did not enter into the councils of its physicians until 1792, when Pinel was made physician at the Bicêtre in Paris, and William Tuke began his merciful work at York, where he shortly afterwards founded the Retreat. Gardiner Hill inaugurated the non-restraintive system at Lincoln Asylum in 1835; and Conolly's (of Hanwell) fluent tongue and silver pen have since fully established the present scientific and humane treatment of the mentally afflicted.

Mr. Greene contrasted the methods of providing for the lunatic populations in Scotland and England, showing that more economical arrangements were made in Scotland. He deprecated the continuous enlargement and increase in lunatic asylums, whilst considerable accommodation was going begging in our workhouses which could well be utilised at a much smaller cost to the ratepayer. Mr. Greene observed that the number of insane were not likely to diminish, and in his opinion the maximum rate of recoveries had been reached. He dwelt on the value of such societies as the After Care Association in diminishing the rate of cases that relapse, and pleaded for more sympathy towards those who had suffered from the terrible affliction of insanity from the public generally. Turning to the nursing of the insane he remarked that the class from which asylum nurses were drawn had greatly improved. At Berrywood the daughters of those belonging to nearly all the professional classes were represented. Here the training was most carefully planned out, and the examinations were real tests of knowledge and efficiency. Every nurse had to serve in the sick ward, which contained as many patients as a small general hospital. Mental diseases came last in the curriculum. The standard of training, he was glad to say, showed a decided upward tendency throughout the Kingdom. Mr. Greene drew attention to the use of the Association of Asylum Workers in spreading knowledge and interest in asylum matters. He dwelt on the advantages offered to those nursing the insane, and advised those desirous of becoming asylum nurses to go nowhere where a nurse was not trained from start to finish in the one institution, and where a good system of training was not provided.

One point amongst many of value in the lecture evoked much interest, and was warmly commended to the attention of the public by Sir Henry Burdett in the discussion that followed. Dr. Greene attributed the large increase in the number of asylums for the insane to the fact that a 4s. capitation grant is handed over to the boards of guardians by the County Council for each patient sent to the county asylum from the union, thus constituting, in his opinion, a direct inducement to the local authorities to get rid of their idiot and imbecile paupers. Dr. Greene suggested that if the County Council were to offer a grant of 2s. per head for every imbecile maintained in his or her local union, the inducement would be for the authorities to make due provision for them at home. Sir Henry Burdett supported this view, adding that the local authorities have plenty of accommodation ready, and that all that is required is proper arrangements for the treatment and supervision of the cases.

### Echoes from the Outside World.

### AN OPEN LETTER TO A HOSPITAL NURSE.

Again the Boers have succeeded in showing their "slimness" just as the last link was being forged in the chain which was to surround them, and have made good their escape. It is believed, however, that it may still be possible to cut off the commandoes retreating from Wepener and Dewetsdorp. General Hamilton, with 18,000 troops, has been making rapid movements, and is expected shortly to come into contact with General Botha, when a big battle is anticipated. Meanwhile Mafeking reports itself "buoyant," and Lord Roberts has written to say that an ample supply of warm clothing has been provided for the men, so that the fear of pneumonia becoming rife, owing to want of sufficient woollen garments, is at an end.

The spell of July weather which so unexpectedly fell to our share in the middle of April had the result of giving most people cold as soon as the thermometer went down again. Spring colds are proverbially difficult to get rid of, which I suppose accounts for seaside places being so much patronised at present. Anyhow, St. Leonards, where I have been trying to court the sunshine and forget the east wind, was particularly full. I have never before been at a health resort during the progress of a big war, and I found the experience a saddening one. The invalids from consumption, rheumatism, and pneumonia form of themselves a sufficiently large army, but just now, when the victims of cold steel are added to the number, a walk by the sea brings many a heartache. A young officer at the hotel, who sat at the next table to us, crept daily into the room assisted by his valet, one leg being perfectly useless. His crutches, so shining and new, told their own tale of how short a time they had been in use, and we learned that the poor fellow was wounded at Spion Kop. Though only twenty years of age, so many of his superiors in command had been incapacitated earlier in the action that he had to lead his men, and even when wounded felt that he could so ill be spared that he went on till he dropped from exhaustion. Then for hours he lay on the ground untended, and for some days it was feared that he would not pull through. Now his boyish brightness has in a large measure returned, but the paralysis—the bullet passed through the stomach, grazing the spine in its course—is very difficult to combat, and foreign cures are to be tried. He was evidently a hero to his two younger brothers as well as to the inmates of the hotel, and had he needed anything, such was our martial ardour, that any of us would have been proud to assist. But he carefully avoided conversation with outsiders, probably because he was afraid of any excitement being followed by a sense of fatigue.

I believe it is a unique experience for a Lord Mayor to have charge of three important Mansion House Funds at the same time. But that is the position in which Sir Alfred Newton finds himself. For the colony suffering from the horrors of war nearly £900,000 has been subscribed; for the vast dependency overwhelmed with famine upwards of £200,000 has been contributed for food; and now that a third disaster, the fire in Canada, has followed close on the heels of the other troubles, it has been decided to open a fund for the relief of the 18,000 persons rendered homeless in Ottawa. Complaints have been made that it was hard to ask the public for more money when they have already freely given. But, as a grateful nation, we could not do less than try to mitigate the distress of a land which so bountifully gave us substantial sympathy when we needed it, not only sending us men, but also money for our widows and orphans. The first use to which the money subscribed—the amount is already over £12,000—is to be put is to provide heads of families with tools and materials for the rebuilding of their ruined homes. This is assuredly helping the sufferers to help themselves. Overcrowding is so great, buildings large enough to accommodate six or seven inmates now sheltering twenty-five to thirty souls, that the work is to be pressed on as much as possible, in the hope of avoiding the outbreak of an epidemic.

The folly of admitting the public to half-finished buildings was demonstrated in Paris on Sunday in a very sad manner. The elevated footway which collapsed at the Exhibition was not being used as a path at the time, although it was to have been tested on the Monday so as to see whether it was capable of withstanding a heavy crowd. Had the public been on, as well as under it, the lives lost would have been far more. As it was, eighteen people have been injured or killed, and many others will probably suffer from shock. The builder of the bridge naturally fails to understand how it could have collapsed in the manner it did, although he appears conscious that the supports which held up the plaster were very slender. The following afternoon a scaffold gave way and killed a couple of workmen, so that it is quite time the demand for an immediate inspection of all structures connected with the Exhibition should be attended to. It is bad enough to accept entrance fees from the public when only half the show is complete; but it is far worse to let them walk into a death-trap. And, apparently, the disasters have in no way been due to undue curiosity or disobedience to warning on the part of visitors, but simply to the desire on the part of the promoters to hurry on matters so that they might the sooner reap a golden harvest. Unless more care is taken, the Exhibition will run the risk of being shunned, and that just now is most undesirable from other than French points of view. The Prefect of Police has very wisely ordered several of the shows to be closed as the safety of the buildings appeared doubtful.

Those of us who have been lucky enough to see "Ecce Homo" and "Christ before Pilate" could not fail to experience a pang of regret upon hearing of the death of the great Hungarian painter. It has been given to few men to conceive and to carry out pictures so instinct with religious feeling, and so realistic in their every detail that they have impressed themselves upon all who have ever studied them in a way never to be forgotten. How much of the man's soul he gave to his work can be judged by the fact that Munckacsy's mental disease dates from the painting of "Ecce Homo." He became so absorbed that he neglected the simplest and most imperative rules of health, having no regular meals, and seldom moving out of his studio even for a breath of fresh air. He worked on there till nothing remained to be added to the masterpiece but the signature. He wrote his name, and as soon as he had done so fell prostrate to the ground, and from that hour he never painted again. He was an artist born, not made. He lost both parents in early infancy, then the relations who sheltered him were all slain by robbers, he alone escaping, and at last he was apprenticed to a joiner. He had enjoyed no education, but his business led him to a college, where he had carpentering to do, and there he taught himself to read and write, studying Schiller later on in his leisure after working fifteen to sixteen hours a day. At last his chance came. He was brought into contact with the painter Szamoscy, and he succeeded in obtaining a few lessons, improving so rapidly that a little later he was capable of painting the portraits of a tailor and his family in return for a new winter overcoat. After he obtained admission to the Paris Salon in 1870 his position was assured, and England and America, as well as the Continental cities, have since vied with each other in crowning him with laurels.

### The Nurses' Bookshelf.

[We invite Correspondence, Criticism, Enquiries, and Notes on Book likely to interest Women and Nurses. Address, Editor, THE HOSPITAL (Nurses' Book World), 28 & 29, Southampton Street, Strand, London, W.C.]

BURDETT'S OFFICIAL NURSING DIRECTORY, 1900. A Directory of Nurses, compiled and edited with the assistance of a small committee of medical men and matrons. By Sir HENRY BURDETT, K.C.B. (London: The Scientific Press, Ltd., Southampton Street, Strand. 5s.)

IT is satisfactory to find that the "Official Nursing Directory" continues to increase in bulk, for this means that the number of nurses who take advantage of an unrivalled channel for making their existence and their qualifications known to the outside world, as well as to the members of the medical profession, is still steadily growing. The volume, of course, contains other features of interest, especially to nurses themselves. It is of great importance to them that they should be fully acquainted with the principal laws affecting their calling; and an intimate knowledge of the general and special hospitals and infirmaries which train probationers cannot fail to be useful. A nurse also often wants to ascertain whether a nursing institution is managed by a committee or by an individual. She should likewise be well informed as to the hospitals and institutions where nurses are employed but not trained; she should be familiar with the names and whereabouts of Colonial, American, and foreign hospitals and nursing institutions; and she should be aware of the existence of all organisations which were founded for the benefit of the profession. On these matters the fullest and most reliable information is provided. But the Directory could be made yet more serviceable and complete by the aid of some of our readers. We are glad to learn from the preface that an article which appeared early in the year in these columns urging upon nurses the necessity, in their own interests, of taking steps to have their names entered in the Directory, had an appreciable effect. In the case of the few matrons who, as the Editor says, will not verify the particulars of training relating to their nurses, it is difficult to suggest a remedy. But as a rule a nurse who wishes her name to be entered in it in the proper manner can get an application form filled in properly by the exercise of a little trouble; and nothing that is not worth a little trouble is of much value to anyone. The Directory, which is now issued for the third time, has obtained the position of a recognised authority, and nurses who are excluded from its pages through thoughtlessness, run the risk of sharing the fate of the unqualified individuals who would, in any event, be denied admission. That is to say, a doctor or any person who requires a trained nurse, and has the Directory for reference, naturally communicates with someone whose antecedents are at hand instead of making inquiries about a nurse whose name is only a matter of hearsay or of indistinct recollection.

### TRAVEL NOTES AND QUERIES.

FINGAL'S CAVE (Penelope).—It is on the small island of Staffa, off the west coast of Scotland, to the west of Mull, and about 10 miles to the north of Iona. No, you cannot live there, but accommodation may be had at Iona very agreeably, and you could go to Staffa by boat in calm weather for sketching purposes. In very rough seas no one could land on Staffa. It is a cruelly rock-bound island, with no sort of bay or harbour.

JERSEY (Impecunious).—It is still cheaper in some respects than England, but not to the extent of 15 or 20 years ago. There are no taxes, which make an agreeable difference; but house rent is not particularly low, and clothing, household goods, &c., are dearer than in England. On the whole, I do not think you would find much difference between Jersey and a provincial town in England.

MALAGA (Perpetual Motion).—I do not think you would like continued residence; six months might be pleasantly spent there, and the rest of the year in more bracing climes. Furnished apartments would be the best. 2. First-class to Royat, £4 8s. 8d.; second class, £3 0s. 9d.

### A Fatal Accident to a Sister at the Belgrave Hospital for Children.

AN accident, causing the death of Miss Beatrice Anne Jefferies, occurred last week at the Belgrave Hospital for Children, Pimlico. It is supposed that Miss Jefferies, in instructing a new probationer how to fill a spirit lamp used for warming the infants' food, had failed to perceive that the flame was not quite extinct. Consequently, when the fresh spirit was added, it burst out again unexpectedly. The probationer's hair then caught fire, and in endeavouring to put it out Miss Jefferies' own clothing became ignited. The flames then got beyond control, and cries for assistance brought the resident house surgeon, the lady doctor, who was also on the premises at the time, and the lady superintendent to the rescue. Miss Milestone, the lady doctor, succeeded in laying Miss Jefferies on the floor, and the flames were speedily extinguished by wrapping her in blankets. Her injuries were at once dressed, but unfortunately she died from the shock. Miss Jefferies, who was only thirty-four years of age, has been in the hospital for the last eight years, and was best known as Sister May. She was a most capable and experienced nurse, and was much beloved by all who knew her. She had received a year's training in the Hospital for Sick Children in Great Ormond Street before taking up work at the Belgrave Hospital; and she had also had some instruction in nursing at Adelaide. She was buried on Wednesday at Nunhead. Great sympathy is felt with all the staff of the hospital. Miss Milestone's hands were badly burned, but the fact only became known after she had done all in her power for Sister May. The hospital was closed for the week, to give the nurses time to recover from the terrible shock and sorrow they sustained before undertaking new cases.

### Appointments.

CHELSEA INFIRMARY.—Miss Annie Rougier has been appointed Home Superintendent, in place of Miss Barton, who has undertaken the care of the Chelsea Convalescent Home at Westgate. Miss Rougier was trained at the National Hospital for the Paralysed and Epileptic, Bloomsbury, and has been for the last two years charge nurse at Chelsea Infirmary.

CROMARTY COTTAGE HOSPITAL.—Miss Ellen E. Wilson has been appointed Nurse Matron. She was trained at St. Marylebone Infirmary. She has since been superintendent nurse at Romford Infirmary, Queen's nurse in Scotland and Ireland, and matron for twelve months at the Ellen Badger Memorial Hospital, Shipston-on-Stour.

THOMAS KNIGHT MEMORIAL HOSPITAL, BLYTH.—Miss Cecil Bell has been appointed Nurse Matron. She was trained at Sunderland Infirmary, and was subsequently charge nurse in both medical and surgical wards in the same institution.

CHELTENHAM GENERAL HOSPITAL.—Miss Gertrude Möller has been appointed Matron. She was trained at St. Mary's Hospital, London, where she subsequently acted as staff nurse for five years, and then as sister-in-charge of the male surgical wards for six.

LAWSON MEMORIAL HOSPITAL, GOLSPIE.—Miss Mackay, formerly matron of the Stranraer Cottage Hospital, has been appointed Nurse Matron of the Lawson Memorial.

### Wants and Workers.

MISS M. E. MAINGAY sends many thanks to those nurses who have so kindly answered her appeal for helping the invalid nurse in furnishing a small bed-sitting-room. Will some others be so good as to contribute a little more, as she has not received quite sufficient to cover the expenses?—Havelet, Florence Road, Boscombe, Hants.

### Receptions to Liverpool Nurses.

BY ONE OF THE GUESTS.

THE Town Hall, of which the citizens of Liverpool are justly proud, presented a very pleasing appearance on the two evenings of April 30th and May 1st, the Lord Mayor and the Lady Mayoress, with characteristic kindness, having arranged two exceedingly pleasant "At Homes" for the nurses of Liverpool. Owing to the fact that only one half of the guests invited could be at liberty at once, some nurses were entertained on Monday and others on Tuesday. About six hundred guests were present on each evening—matrons, nurses, doctors, house doctors of the various institutions and hospitals, the presidents and other honorary officers. The uniforms of all colours contrasted well together, "blues" and "pinks" predominating; the dark blue gowns also looked very well, mingling with the lighter colours effectively. The beautiful appearance of the suite of rooms and staircases was accentuated by the presence of lovely Ascension and arum lilies, and many other splendid plants and flowers. A choice selection of music was played by an orchestra conducted by Mr. Thomas Shaw, in the magnificent large ball-room, and also by the Constabulary Band in the vestibule. Miss Ethel Dewhurst and Mr. Thomas Barlow were most delightful vocalists, and gave the greatest pleasure by their rendering of several favourite songs.

The Lady Mayoress had on a gown of black satin and velvet, trimmed with jet shamrocks; she carried a lovely bouquet of white and yellow flowers. Animated photographs were shown by Messrs. Archer and Sons in the dining-room, photographs of famous generals and recent scenes of the war in South Africa rousing great enthusiasm amongst the audience. Refreshments were served during the evening in the small ball-room. The various hospitals were well represented. Nurses from the Royal Infirmary, wearing their pretty silver medals; the Southern Hospital, the Northern, the workhouse infirmaries, fever hospitals, and many private nurses, all joined together in the dancing which took place from nine till eleven, and appeared to be greatly enjoyed. Perhaps the most conspicuous, and certainly the most interesting uniform was that of the Queen's Nurses, whose bronze badges (the matrons having silver ones) were in evidence. On their left arm was the brassard, on which is inscribed "V.R.I." Many of the guests wore roses. A crowd gathered round the doors of the Town Hall to welcome the coming and to speed the parting nurses, who included at least one stranger from London.

### Minor Appointments.

DUMFRIES AND GALLOWAY ROYAL INFIRMARY.—Miss Catherine J. Spencer has been appointed Charge Night Nurse. She was trained at Charlton Union Workhouse Hospital, and has since been successively superintendent nurse at the Gressenhall Union Workhouse, charge nurse at the Eastern Fever Hospital, Homerton, and private nurse at the Cumberland Infirmary, Carlisle.

BRIGHTON WORKHOUSE INFIRMARY.—Miss Sara B. Boyd has been appointed Charge Nurse. She was trained for three years at the Infirmary, Birmingham, where she did temporary charge work. Miss Boyd holds the L.O.S.

DERBY ROYAL INFIRMARY.—Miss Margaret Brough has been appointed Theatre Sister. She was trained at Bradford, and has since been sister of the women's ward and operating theatre at the General Hospital, Altrincham, Cheshire.

HASTINGS UNION.—Miss Elizabeth Floyd has been appointed Assistant Nurse. She was trained by the Meath Workhouse Attendants' Association, at St. Peter's, Maybury Hill, Woking.

### Everybody's Opinion.

[Correspondence on all subjects is invited, but we cannot in any way be responsible for the opinions expressed by our correspondents. No communication can be entertained if the name and address of the correspondent is not given, as a guarantee of good faith but not necessarily for publication, or unless one side of the paper only is written on.]

### "THE ONE THING NEEDFUL."

"PHYLLIS" writes: Nurse Mary seems not to be aware that one of the consolidated orders is to the effect that prayers shall be read night and morning in each part of the workhouse. If no officer is available a pious inmate may be deputed for the duty. I regret that I cannot give the exact words. Acting on this I have instituted (with the matron's approval) prayers in two workhouse infirmaries. No objection was ever raised by the patients. Roman Catholic nurses have objected on the rather far-fetched ground that the Protestant patients might object to them, and I have had trouble with other nurses, but it is well worth the trouble to gain this point. Patients should be encouraged to express a wish to see a minister, or any particular one, before they are actually dying. This should be sent in writing to the master, who dare not ignore it.

### INEXPERIENCED MONTHLY NURSES.

"A LATE SISTER" writes: The sister who first introduced this subject wrote after some years of experience in private work. Perhaps she did not state clearly about the blanket. She strongly condemns the usual sanitary sheet; it naturally gets saturated at the time of labour, and is difficult, very difficult, to cut into four pieces. Even then, when it cannot be burnt with convenience in the kitchen grate, and methylated spirit is poured upon it and lighted, it smoulders quite as much as it does when burnt, and oh! the awful "choky" smouldering smell which penetrates to the library, dining-room, and bed-rooms. She supposes a careful nurse, one well trained in antiseptics, &c., would naturally fold the sanitary sheet in an old towel and bake it in the oven the day before it was required to ensure it being perfectly free from germs, because the nurse does not know who packed the sheet in paper, or who prepared it. Therefore it is quite as easy to do the same with the old under-blanket. Most nurses know that the inner middle shelf of the oven takes out, so it is quite easy to bake blanket and sanitary towels before using them.

### NURSES AND THEIR CRITICS.

"SISTER MARY" writes: I have been reading with much interest the different criticisms upon nurses, what they should do and what they should not do. May I say a word or two now in their favour? I consider there is far too much morbid sentimentalism about them and their work. One never hears the ways of doctors criticised as those of nurses, and yet their work all leads to the same end, namely, the care of the sick and the dying. A nurse's life is made so excessively hard that one should rejoice rather than condemn the fact that the opportunity of a good dance has been put in her way, be it inside or outside the hospital. Nothing is so beneficial to a hard-working nurse as to carry her right away from the thoughts of her work when off duty. She returns infinitely the brighter and better for it. As to her thinking of her wardrobe when on duty, I don't think those who imagine this really understand what a nurse's life is. Do the critics who draw such hard and fast lines for us realise what a fearful strain the life is? I doubt it. I cannot see why objections should be made to the wearing of uniform at social functions. How many women are there who are not secretly delighted at the attentions of a man in uniform? Yet compare the life-work of a soldier and that of a nurse! If the tender-hearted cannot bear the reminder of a sick-room what agonies they must endure at the sight of a red coat, especially those who have recently lost dear ones at the front! In nine cases out of ten a nurse looks her best in uniform. Were it otherwise, there would probably be no objections raised. If the well-wishers would suppress, instead of excite, criticism, they would be doing a really good work. No one is perfect, and if people would only take themselves to task instead of others the world would be a very different place to what it is.

### For Reading to the Sick.

"MY presence shall go with thee."

Lord, what a change within us one short hour
Spent in Thy presence will prevail to make,
What heavy burdens from our bosoms take,
What parched grounds refresh, as with a shower!
We kneel, and all around us seems to lower;
We rise, and all, the distant and the near,
Stands forth in sunny outline, brave and clear;
We kneel, how weak; we rise, how full of power.
Why, therefore, should we do ourselves this wrong,
Or others—that we are not always strong,
That we are ever overborne with care,
That we should ever weak or heartless be,
Anxious or troubled, when with us is Prayer,
And joy, and strength, and courage are with Thee?
—*Archbishop Trench.*

I am so weak, dear Lord, I cannot stand
One moment without Thee;
But oh! the tenderness of Thine enfolding,
And oh! the faithfulness of Thine upholding,
And oh! the strength of Thy right hand,
That strength is enough for me.
I am so needy, Lord, and yet I know
All fulness dwells in Thee;
And hour by hour that never-failing treasure
Supplies and fills in overflowing measure
My least, my greatest need; and so
Thy grace is enough for me.
It is so sweet to trust Thy word alone;
I do not ask to see
The unveiling of Thy purpose, or the shining
Of future light on mysteries untwining;
Thy promise-roll is all my own,
Thy word is enough for me. —*F. R. Havergal.*

**Reading.**

No one path can lead us so directly towards perfection as a constant abiding in the presence of God. When we realise that we are safe, neither devil nor man can harm us. God has set His temple within our hearts, and if we banish Him thence, what good thing can we hope or look for? All the minor troubles and contradictions of life lose their sting when brought into that Presence. God has promised to fill up whatever is wanting to us with Himself. Is not this a thought which will stay us up under any trouble, and lead us on with rapid steps towards perfection? If friends fail us, or hopes are withered, or toil frustrated, what does it matter if He will fill up the blank? We must dwell more on God, and less on self; we must commune with Him; tell Him freely of our joys and sorrows, our needs and longings. Practically, when so doing, we are making acts of faith, hope, and charity; and as we refer all that concerns us to Him, we go more and more out of ourselves. His will becomes a more prominent feature in our mental horizon; our own troubles and desires sink before it. Thus when harassed or distressed, if instead of dwelling upon our own suffering, or the unkindness or injustice which causes it, we throw ourselves out upon God, letting the outpoor of an over-full heart flow into His Bosom, there will assuredly be a sense of relief, of stayedness, such as nothing else can give. A habit of mind which turns all things to Him is practically dwelling in His continual Presence. Thus in disappointments caused by others, instead of fretting, if we call to mind how often we disappoint God; when friendship or affection fails to give all we need, if we bethink how we fail towards our dearest Friend and Lover, and so on, we shall find the practice (as it is called) of His Presence becoming a reality, and more easy day by day. Then we must take things as they come, thankful alike for what He gives and what He refuses; remembering that we cannot expect always to have sunshine and ease if we are the servants of a suffering Master. If we would tread in His footsteps we must reckon on many a thorn, many a nail, jagged and piercing; many a spear-point, pricking, piercing our hearts; many a season of loneliness, desolation, parching thirst, and weary waiting. But as we learn to see all our own trials in the light of His Presence, as the chord of self melts into the harmony of His heavenly choir, we shall take all these things gladly, and the "not I" of St. Paul will become an increasing reality to us.—*H. Sidney Lear.*

### Notes and Queries.

THE Editor is always willing to answer in this column, without any fee, all reasonable questions, as soon as possible.

But the following rules must be carefully observed:—

1. Every communication must be accompanied by the name and address of the writer.
2. The question must always bear upon nursing, directly or indirectly.

If an answer is required by letter a fee of half-a-crown must be enclosed with the note containing the inquiry.

*District Nurse.*

(44) 1. I am a trained nurse, but have no experience in monthly nursing. Would it be possible, as I am married, to take a district nurse's place, and live at home? 2. What is the meaning of the Holt-Ockley system? Would it require a long and expensive training?—*Anxious H.*

1. It might be arranged under favourable circumstances, as married women often continue private monthly work, and do very well. But it is important that you should be thoroughly trained, and we should advise you, if you can raise the money, to come up to town and train in maternity nursing at one of the hospitals. The City of London Lying-in Hospital, City Road, the British Lying-in Hospital, Endell Street, the East-End Mothers' Home, Commercial Road, E., &c., are all good and cheap institutions. 2. The Holt-Ockley Nursing Association inaugurated a system of supplying homely women to look after the sick poor and to help them in their housework. Apply to the Secretary, 12, Buckingham Palace Road, S.W.

*Middlesex.*

(45) I am anxious to obtain three years' training in a London hospital, and should like to know before applying if the Middlesex ranks with the larger training schools.—*Inquirer.*

Yes.

*Peat Flannel.*

(46) Will you kindly give me the address of the firm who supplies the peat flannel written about in the HOSPITAL?—*Deaconess.*

Messrs. Paté, Burke, and Co., 6, Wool Exchange, London, E.C.

*Masseuse.*

(47) Please inform me if you know of the address of a masseuse in Bath.—*M. D.*

The Secretary, the Society of Trained Masseuses, 12, Buckingham Street, Strand, W.C., will probably be able to recommend one.

*Cheap Accommodation.*

(48) Can you let me know of any establishment or house in which a friend of mine (not a nurse) could live very reasonably but comfortably and within easy reach of the city? I should be greatly obliged. 2. I hope I am writing in time for an answer to appear in your next issue.—*Muriel T.*

You will find a list of boarding homes in London in "The Englishwoman's Year Book" (Messrs. Adam and Charles Black). 2. We are obliged to print our replies in rotation because of the pressure on our space.

*Eczema.*

(49) Since August I have been troubled with a rash on my face which the doctor tells me is a mild form of eczema caused by indigestion. It has twice been cured, but its reappearance troubles me as I have set my heart on being trained as a nurse. I fear it may interfere with my prospects of entering a hospital. Would you kindly tell me if there is a permanent cure?—*A. M. S.*

We cannot prescribe or advise.

*Leprosy.*

(50) Would you kindly inform me if there is a hospital for leprosy in England or if there is one on the Continent for English people?—*Nita (Manchester).*

There is now no hospital in England especially for leprosy; those devoted to this disease in olden time have been converted to other uses. Nor is there any that we know of on the Continent exclusively for the accommodation of English people. A case of leprosy would, however, be treated at any of the hospitals for skin diseases. The Manchester and Salford Hospital for Skin Diseases, Quay Street, is near you.

*Holiday.*

(51) What is the usual length of holiday given to district nurses?—*Nurse Jane.*

That is entirely a matter of private arrangement between the committee and the nurse. Holidays for nurses range very generally from three to five weeks per annum.

*Convalescent Home.*

(52) Could you kindly inform me of any convalescent home for gentlewomen where one could be received who occasionally has attacks of asthma? She could pay from 15s. to 20s. a week.—*A. C. S.*

St. Bernard's Invalid Gentlewomen House, 106, Lansdowne Place, Brighton, receives at the terms you state. Also Yorkshire Convalescent Home for Ladies, St. Martin's Lodge, South Cliff, Scarborough; and Convalescent Institute for Invalid Ladies, Erith House, Torquay. Or you can consult Burdett's "Hospitals and Charities," where you will find a long list.

*Standard Books of Reference.*

"The Nursing Profession: How and Where to Train." 2s. net.
"The Nurses' Dictionary of Medical Terms." 2s. 6d. net.
"Burdett's Series of Nursing Text-Books." 1s. each.
"A Handbook for Nurses." (Illustrated.) 5s.
"Nursing: Its Theory and Practice." New Edition. 3s. 6d.
"Helps in Sickness and to Health." Fifteenth Thousand. 5s.

All these are published by THE SCIENTIFIC PRESS, Ltd., and may be obtained through any bookseller or direct from the publishers, 28 & 29, Southampton Street, London, W.C.

